# Brazilian caregivers’ conception on child bullying

**DOI:** 10.1186/s41155-018-0113-0

**Published:** 2018-11-26

**Authors:** Laila Akerman, Juliane Callegaro Borsa, Ilana Landim, Bheatrix Bienemann

**Affiliations:** Department of Psychology, Pontifícia Universidade Católica do Rio de Janeiro, Rua Marquês de São Vicente, 225, Prédio Cardeal Leme, room 201, Rio de Janeiro, Brazil

**Keywords:** Bullying, Children, Parents, Caregivers, Iramuteq

## Abstract

**Background:**

Bullying is a complex social phenomenon, which is common in peer relationships and is influenced by different individual and contextual characteristics. Despite broad knowledge on the importance of the family for children’s development, many studies about bullying neglect the family’s active role. In that sense, investigating caregivers’ conception about bullying can be an important strategy to promote effective interventions. The objective in this study was to analyze the caregivers’ conception on the phenomenon of bullying, specifically regarding its occurrence, motivations, and risks for the children’s development, and verify if this conception is consistent with the findings of the international literature. The study participants were 401 caregivers (77.1% were mothers) of children in elementary education at Brazilian schools. An online questionnaire was used with closed questions and an open question on what the caregivers considered bullying. The data were analyzed based on descriptive statistics and quantitative textual analysis.

**Results:**

Caregivers have good knowledge on signs and forms of coping with bullying. On the other hand, they tend not to recognize their children as potential aggressors and do not mention the family’s role as a risk factor for the occurrence of this type of problem.

**Conclusions:**

The results allowed us to understand what Brazilian caregivers think about bullying and how they act or would act towards situations of bullying and reveals a relevant gap on this comprehension.

## Introduction

Aggressive behaviors refer to a variety of intentional actions aimed at impairing or causing physical or psychological harm to an individual or a group of people (Berkowitz, 1993; Coie & Dodge, 1998). In childhood, these behaviors can target family members, teachers, peers, animals, or objects (Borsa & Bandeira, 2014) and be manifested in different ways, including physical assaults, such as kicking, pushing, beating, and/or verbal aggression, such as screaming or offending (Björkqvist, 1994; Dodge & Coie, 1987). The literature also suggests different subtypes of aggressive behavior, depending on the underlying motivation (Cooley & Fite, 2016). For example, reactive aggressive behaviors refer to actions that occur in response to a perceived provocation or threat, while proactive aggressive behaviors refer to an action motivated by anticipated rewards (Crick & Dodge, 2018; Dodge & Coie, 1987). Gender differences are also observed in the manifestations of aggressive behavior in childhood: boys seem more inclined to manifest aggression of the physical type, while girls manifest relational aggression more frequently (Archer, 2004; Crick, Casas, & Ku, 1999; Olweus, 1997).

The aggressive relational behavior aims to socially harm a friend or colleague through strategies such as defamation, isolation, and provocation (Crick & Grotpeter, 1996). Aggressive relational behavior is more frequent from the middle of childhood until adolescence, a period when interpersonal relationships become more intense (Dodge, Coie, & Lynam, 2006). A subtype of aggressive relational behavior common in childhood, especially in daily school life, is bullying (Borsa, Petrucci, & Koller, 2015). Bullying is defined as systematic and intentional aggressive behaviors occurring in a context of clear power imbalance between the aggressor and the victim (Olweus, 1997, 2013).

Bullying is characterized by the presence of three criteria that differentiate it from other types of aggressive behaviors in childhood, such as intentionality of the act, continuity and systematization of aggressions, and an imbalance of power between aggressors and victims (Olweus, 1997, 2013). This asymmetry of forces may manifest itself in different ways: the target of bullying may be or perceive itself as physically or psychologically weaker than the aggressor, and there may also be a numerical difference between the aggressors and the victim. Another type of asymmetry of forces can be established when the aggressor is difficult to identify or confront, which can occur when the victim is socially excluded from the group, when the victim is badly spoken of “behind the back” or when the victim receives anonymous messages (Olweus, 1997, 2013). It is important to mention that bullying occurs most of the time without there being an apparent provocation by the victim (Olweus, 2013).

Bullying is a type of behavior commonly practiced in the school environment, a space conducive to the development of social skills and also to the emergence of interaction difficulties among peers (Borsa et al., 2015). In addition, the virtual space has become a common stage for the occurrence of cyberbullying, as the personal attacks performed through interactive technologies are called in the literature (Kowalski & Limber, 2013; Modecki, Minchin, Harbaugh, Guerra, & Runions, 2014).

It is important to emphasize that bullying is observed in different cultures, according to research conducted by Craig et al. (2009), with 202,056 young people aged 11 to 15 years from 40 different countries, where 26.9% of the participants reported some type of involvement in the dynamics of bullying. In the same sense, the meta-analysis conducted by Modecki et al. (2014), considering samples from 80 studies in English, identified a prevalence rate of 35% of bullying among young people aged 12 to 18 years. In Brazil, the National School Health Survey, conducted in the five regions of the country with ninth-grade students, indicated that 7.6% of male students and 7.3% of female students reported being frequently bullied. In addition, 24.2% of male students and 15.6% of female students said they practiced bullying against their colleagues (Instituto Brasileiro de Geografia e Estatística (IBGE), 2015).

Bullying is a group phenomenon (Juvonen & Graham, 2014; Olweus, 1993) and, in this relational dynamics, different roles can be identified: aggressors (who often assume the leadership position), followers (boosters of aggressions), witnesses (those who witness aggressions), and victims (who, in addition to suffering from direct aggression, may also be excluded from social interactions) (Olweus, 1993; Salmivalli, Lagerspetz, Björkqvist, Österman, & Kaukiainen, 1996). In addition, there are victims who also act as aggressors (Salmivalli et al., 1996). Studies indicate that bullying can harm the victims and perpetrators’ physical and mental health and quality of life (Takizawa Maughan, & Arseneault, 2014; Wolke & Lereya, 2015). Bullying victims are likely to have high levels of anxiety and depression in childhood and adolescence, suicidal ideas (Fisher et al., 2012; Takizawa et al., 2014), psychosomatic complaints (Gini & Pozzoli, 2013; Wolke & Lereya, 2015), and difficulties in school (Hammig & Jozkowski, 2013; Wolke & Lereya, 2015). Damage to perpetrators includes the increased risk for long-term involvement with violence (Bender and Losel, 2011; Wolke & Lereya, 2015) and substance abuse (Fite, Schwartz, & Hendrickson, 2012; Wolke & Lereya, 2015).

From a bio-ecological perspective, bullying can be understood as a complex social phenomenon, influenced by different characteristics of the individual, family, school, and society in general (Barboza et al., 2009; Borsa et al., 2015). Although there exists considerable knowledge about the importance of family for human development, especially in childhood, many studies on bullying neglect the effective participation of mothers, fathers, and caregivers (Borsa et al., 2015; Sawyer, Mishna, Pepler, & Wiener, 2011). The family may exert significant influence in the manifestation of bullying in childhood and adolescence (Shakoor et al., 2011). Specifically, the way parents or caregivers understand and treat bullying can influence the way in which they exercise coping strategies in view of the signs of their children’s involvement in this type of conflict (Atlas & Pepler, 1998; Sawyer et al., 2011).

Qualitative study centered on the parents’ perspective suggests that most of the parents were able to define bullying in a way that was consistent to the literature, including a reference to a power imbalance inherent in bullying situations (Sawyer et al., 2011). Nevertheless, other studies indicate that several parents do not consider indirect relational aggression as bullying and struggle to differ bullying from teasing (Harcourt, Jasperse, & Green, 2014; Mishna, Pepler, & Wiener, 2006). A systematic review identifies a wide range of strategies used by caregivers or parents for coping with bullying. Those strategies primarily involve the victim or the victim’s family, rather than the aggressor or their family. Some parents also approach child’s school in order to manage the situation or use specific strategies in response to bullying, like enhancing their child’s ability to handle the bullying on their own, for instance (Harcourt et al., 2014).

Many children do not comment on bullying with caregivers, ashamed to report the experience or due to a lack of confidence on how to manage the situation (Sawyer et al., 2011). The child’s difficulty in dealing with interpersonal conflicts may require effective intervention by parents or responsible caregivers (Atlas & Pepler, 1998; Rigby, 2008). In this sense, investigating what caregivers think about bullying can be an important strategy to promote effective interventions, aiming to reduce aggressive behaviors in childhood, as the results of interventions are better when caregivers are included in the process (Landim & Borsa, 2017). Few studies in Brazil aimed to assess caregivers’ conception on bullying (Borsa et al., 2015). Therefore, the objective of this study is to analyze the caregivers’ conception on the phenomenon of bullying, specifically in relation to their occurrence, motivations, and developmental risks, and verify if Brazilian caregivers’ conception is consistent with the findings of the international literature on bullying.

## Method

### Participants

The study participants were 401 caregivers of children in the first to fifth year of elementary school, from public and private Brazilian schools. The participants’ mean age was 39.6 years (SD = 8.6). Regarding sex, 343 (85.5%) was declared as women and 58 (14.5%) men. As to the degree of kinship with the child, 309 (77.1%) were mothers, 38 (9.5%) fathers, and 54 (13.5%) others (grandparents, uncles, and siblings). The mean age of the children was 8.93 years (SD = 2.2), being 218 (54.4%) male. Regarding the educational level of the child’s father (or caregiver), among the respondents, 18 (5.6%) have not completed elementary school, 16 (5%) have completed elementary school, 23 (7.2%) have not completed high school, 55 (17.1%) have completed high school, 55 (17.1%) have not completed higher education, 73 (22.7%) have completed higher education, 7 (2.2%) have not completed postgraduate, and 74 (23.1%) have completed postgraduate studies level. Regarding the educational level of the child’s father (or caregiver), among the respondents, 7 (2.2%) have not completed elementary school, 10 (3.1%) have completed elementary school, 5 (1.5%) have not completed high school, 45 (13.8%) have completed high school, 53 (16.3%) have not completed higher education, 86 (26.5%) have completed higher education, 13 (4%) have not completed postgraduate, and 106 (32.6%) have completed postgraduate studies level.

### Instruments

The data were collected through a structured questionnaire with closed questions about parents’ conception regarding bullying, specifically built for this study. The content of the questions was formulated based on the national and international literature on bullying and its relation with different variables of the family context. The content of the questionnaire was previously evaluated by expert judges in Child and School Psychology. Afterwards, the content of the instrument was also evaluated by two pairs of parents who reported on the comprehension and clarity of the items. In the questions, the caregivers’ conception on general bullying situations (not specifically involving their children), the strategies they use for prevention and intervention in case of bullying, and how they perceive bullying situations with their children were investigated. “What are the types of aggression occur in the context of bullying?”, “What are the characteristics of the child that facilitates their involvement as a victim of bullying?”, “What are the characteristics of the child that facilitate their involvement as an aggressor?”, “What are the consequences of bullying for the victim?”, “Do you think bullying should be punished?”, “How do you think the child should react when he or she is a victim of bullying?”, “Who do you think is the responsibility of preventing bullying?”, and “How do you think caregivers should react when their child is aggressor in bullying situations?” are examples of questions that composed the questionnaire.

In order to avoid participants’ dropping out during the completion of the questionnaire, caregivers could answer each of the questions or not. In addition, more than one answer option could be chosen for each question. Thus, the frequencies and percentages presented refer to the total number of answers provided and not to the absolute number of respondents. In addition to the closed questions, at the end of the questionnaire, an open-ended question was asked, “What do you mean by bullying?”, which could be answered without character limits. A sociodemographic questionnaire with closed questions was also used, aiming to obtain information from the participants and their families.

### Data collection procedure

The study was approved by an Ethics Committee (CAAE: 05118412.6.0000.5334), and all ethical issues were ensured according to Resolution 466/2012, Brazilian Ministry of Health, and the Declaration of Helsinki. The data were collected virtually, via an online survey software. Participants were recruited through social networks using the snowball technique (Patton, 1990). They were informed of the voluntary and confidential nature of their participation, as well as the objectives, risks, and benefits involved in the research. The criteria for inclusion were to be over 18 years of age, to manifest the free will to participate in the research, to sign the informed consent form, to be Brazilian, and to have at least one child regularly enrolled in elementary school. There was no exclusion criterion, so that all protocols duly completed were considered for analysis.

### Data analysis procedure

First, the information from the structured questions of the sociodemographic questionnaire and the bullying questionnaire were analyzed using descriptive statistics using SPSS (Statistical Package for Social Sciences) software version 17.0 (SPSS Inc., Chicago, IL, USA). The objective of these analyses was to evaluate both the respondents’ frequency and answers to each of the relevant questions in order to meet the objective of this study.

Next, the textual content of the answers to the open question in the questionnaire “What do you understand by bullying?” was analyzed using the R and python language-based software IRAMUTEQ - *Interface de R pour analyses Multidimensionnelles de Textes et de Questionnaires* (Marchand & Ratinaud, 2012). This R Interface permits quantitative analyses of textual data, generating classes of contents deriving from the relationship among the vocabularies that are present. The Descending Hierarchical Classification (DHC) was analyzed to understand, from the text corpus, the lexical roots and what the classes are inserted in (Camargo & Justo, 2013).

The textual corpus was divided into 294 text segments (TSs), which were characterized by research variables (respondent’s sex, income, sex of the child, if they had already been victims of bullying as a child, if they had already practiced bullying as a child), aiming to identify if these variables relate to different conceptions of bullying. Regarding the criterion used for the grouping of the information obtained through the DHC, the software groups the elements into their respective classes through mathematical criteria. It analyzes the frequency of the element in relation to the mean number of occurrences in the corpus, as well as the association with the class by the chi-square coefficient equal to or greater than 3.84 (error margin < .05 and degree of freedom = 1) (Ratinaud & Marchand, 2012). In order to identify the co-occurrences and the connection between words, a similarity analysis was also carried out, which is based on graph theory and helps to identify the representation structure (Camargo & Justo, 2013, Ratinaud & Marchand, 2012).

## Results

Initially, the participants’ knowledge about bullying that did not directly involve their children was investigated. Regarding the frequency, 42.7% (*n* = 134) of the responses consider that the cases of bullying are increasing and 51% (*n* = 160) consider that bullying has not changed in frequency and intensity. Only 1% (*n* = 3) of the answers consider that cases of bullying are decreasing. The most reported aggressions of the context of bullying were of the relational type (49.6%, *n* = 295), verbal (46.2%, *n* = 275), and physical (38.7%; *n* = 230). Among the characteristics of children that facilitate their involvement as victims of bullying, most responses considered physical appearance (43.9%, *n* = 261), shyness (41.3%, *n* = 246), and unpopularity (39%; *n* = 232). Among the characteristics of the children that facilitated their involvement as aggressors, aggressiveness (43.2%, *n* = 257), popularity (29.7%, *n* = 177), and socioeconomic status (28.3%; *n* = 168) were the most cited.

In relation to the consequences of bullying for the aggressors, the most cited characteristics were the popularity in the group of friends (24.5%, *n* = 146), difficulties in family relationships (23.7%, *n* = 141), and leadership development in the group (23.2%, *n* = 138). As for the consequences of bullying for victims, the most cited were psychological difficulties (46.4%, *n* = 276), relationship difficulties with colleagues and friends (44.7%, *n* = 266), and physical health problems (41.7%, *n* = 248). In addition, 97.5% (*n* = 281) of the answers consider that bullying is very or extremely harmful to the victim and 70.2% (*n* = 202) affirm that bullying is very or extremely harmful to the aggressor. As for how the children should react when victims of bullying, the respondents believe that they should tell their parents (47.9%; *n* = 285), the teachers (44%; *n* = 262), and the school principal/coordinators (38.2%; *n* = 227).

Regarding the sex of those involved, 61.5% (*n* = 179) of the responses considered that both boys and girls practice bullying to the same extent, 33.3% (*n* = 97) consider that boys practice more bullying, and only 5.2% (*n* = 15) girls. On the other hand, 69.4% (*n* = 202) of the answers indicate that both boys and girls are victims of bullying. As to age, 52.6% (*n* = 153) of the answers indicate that older children practice bullying most, 44% (*n* = 128) believe that the practice is independent of age, and 3.4% (*n* = 10) believe that bullying is more practiced by younger children.

Regarding prevention, 98.6% (*n* = 283) of the responses identified that it is necessary to prevent bullying and that this should be the parents’ (43.4%, *n* = 258), the school’s (39.5%, *n* = 235), and the teachers’ (39.2%, *n* = 233) responsibility. In addition, education and family values (45.7%, *n* = 272) and communication between parents and children (44.5%, *n* = 265) were considered to be the family characteristics that most contribute to bullying prevention. In addition, among the responses, 86.3% (*n* = 246) considered it important to punish bullying and that this should be the school’s (30.9%, *n* = 184) and the parents’ responsibility (23.4%; *n* = 139).

Regarding how caregivers should react when the child practices aggression, 46.4% of the answers (*n* = 276) refer to the importance of talking with their child about what happened, 44.4% (*n* = 264) consider it important to talk to the school or teacher, and 33.3% (*n* = 200) find it important to talk to the parents of the victimized child. When the child is the victim of bullying, 46.2% (*n* = 275) of the respondents find it necessary to talk to the child about the incident, 45.4% (*n* = 271) believe that parents should talk to the school or teacher, and 35.5% (*n* = 211) state that parents should talk to the caregivers responsible for the offending child. Among the study participants, 70.7% (*n* = 200) believe that there should be a law to punish situations of bullying, but 89.7% (*n* = 253) do not know of any anti-bullying law in Brazil.

Regarding the bullying situations the children experienced, 82.5% (*n* = 231) of the caregivers said that their child had already witnessed situations of bullying and 62.1% (*n* = 174) reported that their child had already been a victim of bullying at school. Nevertheless, 83.9% (*n* = 235) of caregivers affirmed that their child never practiced bullying. When questioned about the child’s reaction when witnessing bullying at school, 22% (*n* = 131) of the responses indicate that the child witnessed and sought help from third parties (such as teachers and principals), 16.1% (*n* = 96) indicate that the child witnessed and defended the victim, 11.1% (*n* = 66) indicated that the child only witnessed but did not participate in the aggression, 4.4% (*n* = 26) indicate that the child witnessed and was amused by the situation, 2% (*n* = 12) indicate that the child witnessed and ignored the situation, and 0.3% (*n* = 2) indicate that the child witnessed and encouraged the abuser.

When questioned about what they would do if the child were bullied, 44.9% (*n* = 267) of the respondents stated that their reaction would be to contact the school or the aggressor, 44% (*n* = 262) would talk to the child about what happened, and 27.9% (*n* = 166) would seek professional help. Only 2.4% (*n* = 14) of them would tell the child to “pay back in his own coin.” If their child reported bullying, 44.7% (*n* = 266) of the respondents would choose to talk to the child about what happened, 33.6% (*n* = 200) would contact the school or the teacher, and 32.9% (*n* = 196) would seek external professional help. If the child reported witnessing bullying among peers, 40.8% (*n* = 243) of the respondents would talk to the child about the incident and 40.5% (*n* = 241) would contact the school or the teacher. The vast majority of responses identified, 92.1% (*n* = 257), reported that the children would tell if they witnessed bullying. Furthermore, 87.1% (*n* = 243) of respondents reported believing that the children would tell them if they were victims of bullying at school, while 57.7% (*n* = 161) reported that the children would tell them if they were bullying at school.

**Fig. 1:**
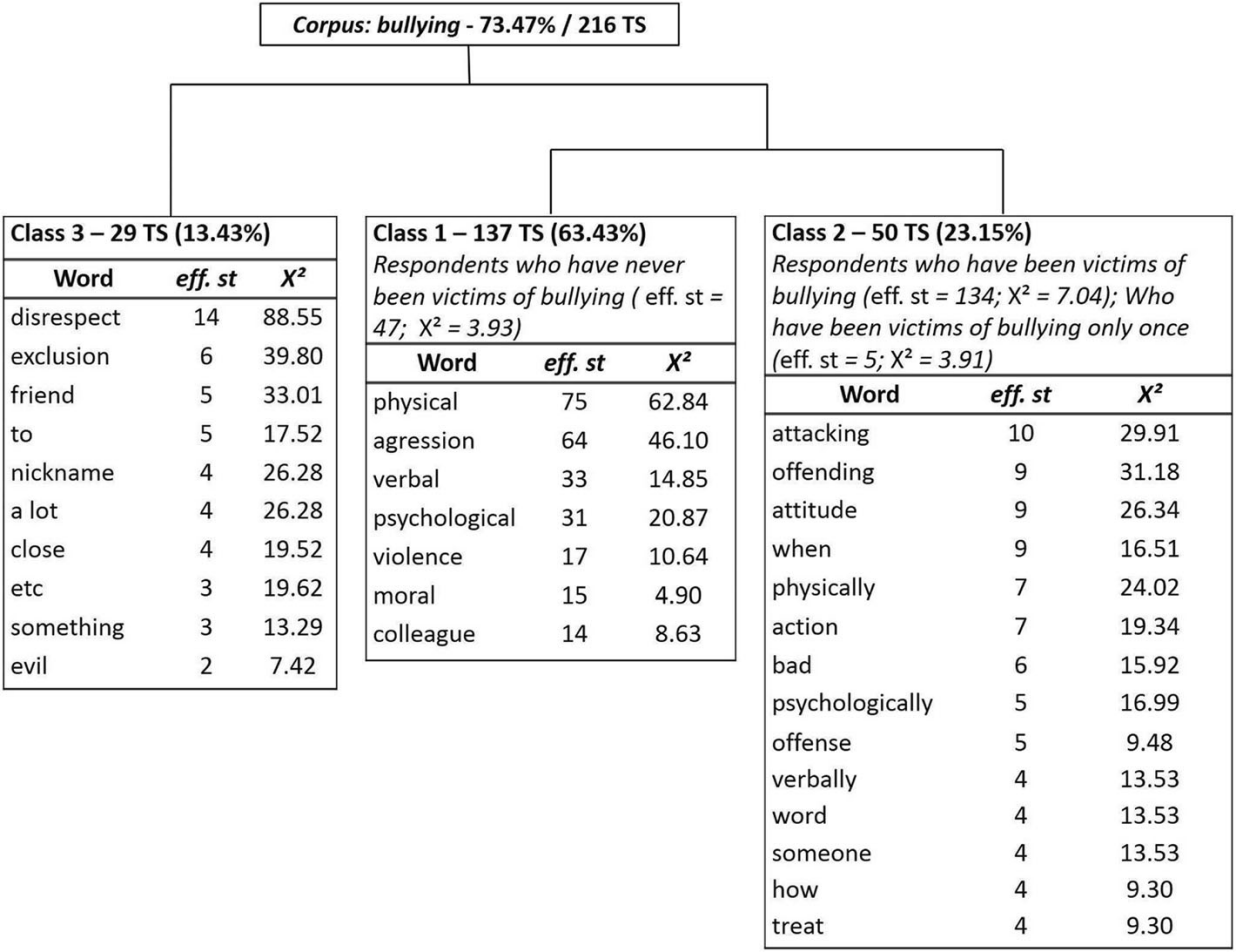
Tree diagram of Descending Hierarchical Classification of the corpus <<What do you consider as bullying>>. Legend: *Note. TS* text segments, *eff. st* word frequency by text segments

The answers to the question “What do you mean by bullying?” were analyzed by means of a Descending Hierarchical Classification (DHC). For this, the set of answers (texts) constituted a corpus of analysis submitted to the program (Camargo & Justo, 2013). In this case, this DHC is a textual set focused on a theme, the understanding of bullying for caregivers. In this sense, the textual corpus was divided into 294 text segments (TSs), listing 779 words that occurred 2885 times (average occurrence per segment = 9.81). The DHC retained 73.47% of total TSs, generating three classes (Camargo & Justo, 2013; Ratinaud & Marchand, 2012). As presented in Fig. 1, at first, the bullying corpus was divided into two subcorpora and, then, the subcorpus on the right was divided in two, obtaining classes 1 and 2. In the figure, the words “physical,” “aggression,” “verbal,” “psychological,” “violence,” “moral,” and “colleague” appear in class 1. The words “attacking,” “offending,” “attitude,” “when,” “physically,” “action,” “bad,” “psychologically,” “offense,” “verbally,” “word,” “someone,” “how,” and “treat” appear in class 2. Finally, the words “disrespect,” “exclusion,” “friend,” “to,” “nickname,” “a lot,” “close,” “etc,” “something,” and “evil” appear in class 3.

Class 1, composed of 137 TSs (63.43%), is characteristic of people who have never been victims of bullying. Class 2, composed of 50 TSs (23.15%), is characteristic of caregivers who affirmed having been victims of bullying. The two classes share statistically significant variables (*p* < 0.005), such as the variables associated with a representation of bullying as <physical>, <aggression>, <psychological>, and <verbal>. Thus, these are the main variables that organize the defining conceptions of bullying in the perception of both the caregivers who have never been victims of bullying and those who have once or more. Nevertheless, in class 2, other variables were significant, such as <offend>, <attitude>, <action>, <evil>, and <word>, for example. Class 3, composed of 29 TSs (13.43%), was not significantly associated with any characteristic.

**Fig. 2:**
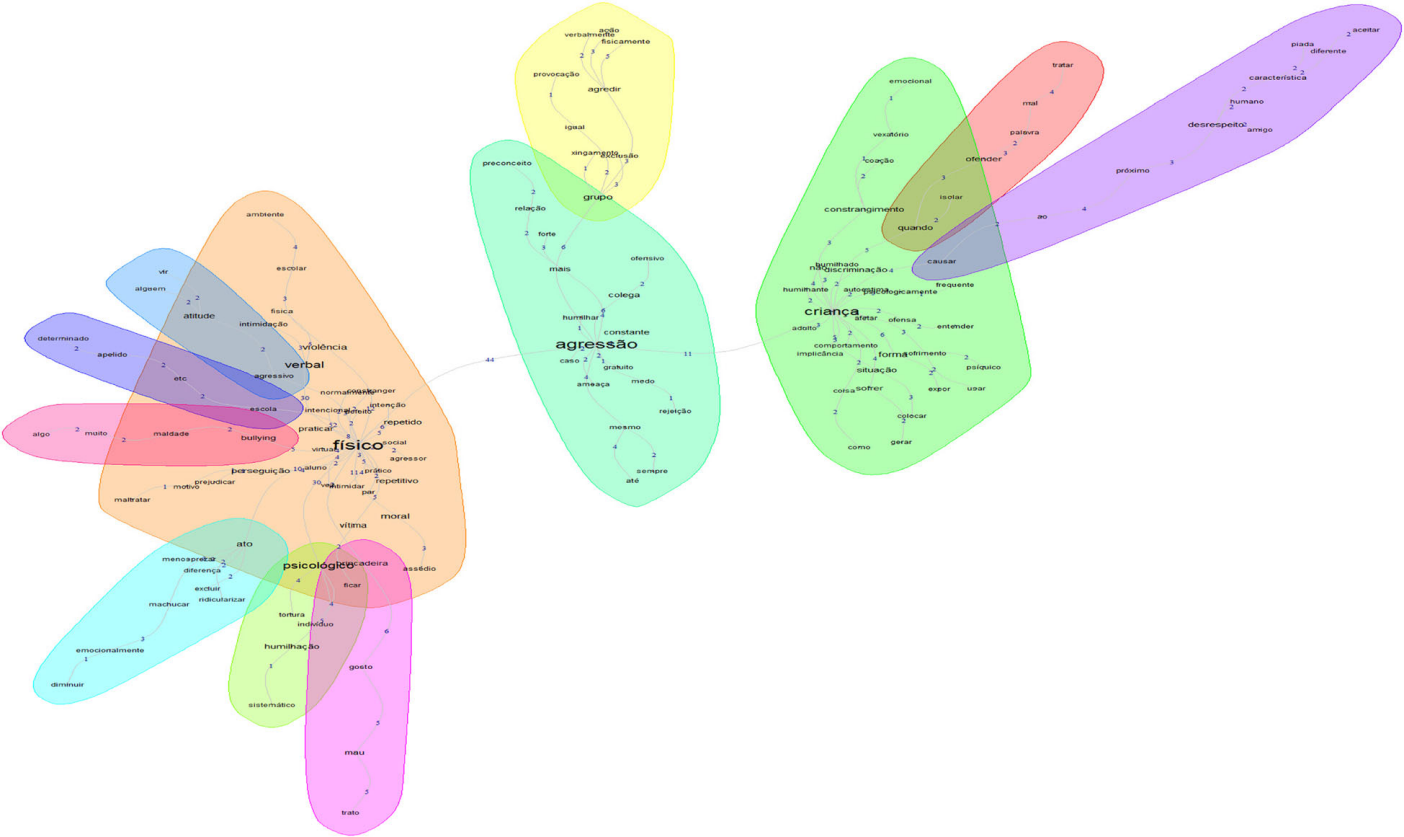
Similarity analysis of the corpus

Another type of analysis performed is that of similarity. Based on the graph theory, it permits identifying co-occurrences of words, demonstrating the connection between words and identification of the content structure of the textual corpus (Marchand & Ratinaud, 2012). In this sense, in a complementary analysis of similarity, according to Fig. 2, it can be observed that the terms “aggression,” “physical,” and “child” organize the concept of bullying. The term “aggression” is associated with the terms “group,” “relationship,” “rejection,” “exclusion,” “cursing,” among other terms. The term “physical” is associated with the terms “psychological,” “verbal,” “harassment,” “moral,” “embarrassment,” “persecution,” “joke,” among other terms. The term “child”, associated with the term “aggression”, was related to the terms “implication,” “constraint,” “self-esteem,” “discrimination,” and “emotional,” as shown in Fig. 2.

## Discussion

This study aimed to investigate caregivers’ conception, based on an understanding about the definition of the phenomenon, to an investigation about how to react to a bullying situation. Regarding the answers to the question “What do you mean by bullying?”, the similarity analysis of the textual corpus generated by the R interface (IRAMUTEQ) identified that the terms “aggression,” “physical,” and “child” are organizers of the caregivers’ concept of bullying, revealing the concept of aggressiveness as a nuclear theme related to the phenomenon. This general conception is, partially, similar to Olweus’(1997, 2013) definition of bullying, the precursor of research on the subject. In addition, the Descending Hierarchical Classification (DHC) analysis allowed to identify that although a significantly shared opinion about bullying is structured, people who were victims (class 2), presented more specific descriptors related to verbal and psychological aggression, such as <offend> and <word>. The presence of the descriptor <evil> could be related to an emotional response evoked by the victimization experience (Sampaio et al., 2015).

One of the results is that most of the participants consider that the frequency and intensity of the bullying did not change, but that the name did not exist before. This data may indicate an understanding of the caregivers that the manifestation of bullying is a historically present and silenced practice, alerting to the importance of conducting research and interventions that aim to manage the perpetuation of bullying in childhood, especially in the Brazilian context, where the theme gained relevance only in the first decade of the twenty-first century (Bandeira & Hutz, 2014). In addition, while recognizing that bullying practices are historically present, the issue’s major role is related to social changes regarding the guarantee and extension of rights to children, so that bullying is seen as a violation of the child’s health and integrity, turning into a violence to be fought, instead of a child’s play (Kolstrein & Jofré, 2013).

Regarding the frequency of bullying, a significant number of respondents mentioned that it is increasing, as opposed to the study by Rigby and Smith (2011), which identified that the frequency of bullying has actually decreased, especially due to the efforts of intervention and prevention programs. In the Brazilian context, the high prevalence of bullying (Instituto Brasileiro de Geografia e Estatística (IBGE), 2015; Malta et al., 2010) is related to the severity and social impact the problem of violence causes in the Brazilian society (Malta et al., 2010). In addition, there is the precariousness of intervention and prevention strategies. This is due to the fact that many intervention programs to reduce aggressive behaviors generally do not meet the requirements of appropriate evaluation of effectiveness, often related to the use of exclusive self-report instruments and techniques, lack of consistency in the pre- and post-evaluation, and the absence of follow-up (Landim & Borsa, 2017).

Among the types of aggressions the participants mentioned, relational aggressions (humiliation, gossip, exclusion, etc.) were the most reported, followed by verbal and physical aggressions. This data is consistent with the literature, as verbal aggressions are more common than physical aggressions in bullying, especially as age advances and verbal language improves (Bandeira and Hutz, 2012, 2014; Dodge et al., 2006; Olweus, 1993; Terroso, Wendt, Oliveira, & Argimon, 2016). Nevertheless, although part of the participants’ acknowledge that relational aggressions are the most frequent, physical aggression is the main type in the collective imaginary concerning bullying and the target of caregivers’ major concern (Borsa et al., 2015; Sawyer et al., 2011). It is important to mention that who has contact with children involved in situations of bullying should be capable of observing the effects of this practice on child development (Shakoor et al., 2011).

Caregivers identified that physical appearance is the main feature that facilitates the child’s involvement as a victim of bullying, followed by shyness and unpopularity. This conception is in accordance with other studies that identify characteristics that favor victimization, including anxiety (Pabian & Vandebosch, 2016), low self-esteem (Tsaousis, 2016), loneliness, lack of close friends (Acquah, Topalli, Wilson, Junttila, & Niemi, 2016), and physical characteristics, such as appearing to be overweight or very skinny (Reulbach et al., 2013). Another factor found in the literature as a trigger of bullying is the presence of mistreatment and domestic violence (Bowes et al., 2009). The caregivers of the sample did not present these factors, not mentioning the influence of the family in the children’s experiences of victimization. This data can be justified by a possible method bias, as a self-report questionnaire was used in this research, whose responses may have been influenced by social desirability. Studies indicate that dissatisfaction with body image is a variable strongly associated with victimization in bullying situations and is associated, albeit to a lesser extent, with aggression (Levandoski & Luiz Cardoso, 2013; Rech, Halpern, Tedesco, & Santos, 2013). In Levandoski and Luiz Cardoso (2013)’s study, it was identified that both victims and aggressors would like to be physically “bigger,” for example, which may be related to the growing appreciation of a muscular type, especially during adolescence (Brixval, Rayce, Rasmussen, Holstein, & Due, 2012). The same study found, however, that the offenders are generally more satisfied with their own body image than the victims. Yet, overweight children are more vulnerable to bullying than their normal weight counterparts.

Aggressiveness, popularity, and socioeconomic condition were considered the characteristics that most facilitate the involvement of the child as an aggressor. This conception of the caregivers is also consonant to what the literature points out about the aggressors in bullying. Studies indicate that aggressors have a more positive attitude towards violence, including impulsiveness and satisfaction in being in control, often becoming aggressive adults (Olweus, 1997; Wolke & Lereya, 2015) and are generally considered “popular” by their peers, presenting sociocognitive skills that reinforce the position of aggressor, such as the ability to anticipate peer reactions, elect a vulnerable victim, and use aggression in their favor (Almeida & Lisboa, 2014; Terroso et al., 2016).

Participants listed that popularity in the group of friends, difficulty in relating to family members, and developing leadership in the group are the most frequent consequences of bullying for perpetrators. In other words, it is observed that caregivers perceive both positive and negative consequences for the aggressors, which is consistent with the literature (Bender and Losel, 2011; Fite et al., 2012; Wolke & Lereya, 2015). Among the gains, studies indicate that aggressors feel powerful and confident and with high self-esteem (Olweus, 1997, 2013). As a negative consequence, in line with the participants, family conflicts may be related to both the prediction and the consequences of aggressive behavior in childhood, the family being an essential variable in the children’s development, being the scenario for learning from the imitation of behaviors (Almeida, Silva, & Teodoro, 2014). It is also important to note that, despite the recognition that engaging in bullying results in gains for perpetrators, most participants consider bullying as very or extremely harmful to the perpetrator. Studies show negative short-term consequences for perpetrators, such as a subjective feeling of post-attack malaise and, in the long term, including increased involvement in lifelong violence (Bender and Losel, 2011; Wolke & Lereya, 2015).

Research participants observed negative consequences for the victims of bullying, especially psychological difficulties, relationship difficulties with colleagues and friends, and physical health problems. The literature broadly corroborates this data, pointing out social damages and impairments in the physical and mental health of victims of bullying (e.g., Brendgen & Poulin, 2018; Gini & Pozzoli, 2013; Hammig & Jozkowski, 2013; Wolke & Lereya, 2015). Almost all of the participants consider bullying as very or extremely harmful to victims, alerting them to a certain level of awareness about a phenomenon that was erroneously considered, for a long time, as a natural rite of passage in childhood (Wolke & Lereya, 2015). It is observed that the caregivers do not always refer to the fact that bullying is a public-health issue associated with negative physical and psychological effects, especially in the long term. Perpetrators and victims of bullying may present depressive symptoms (Fisher et al., 2012; Wolke & Lereya, 2015) and, in extreme cases, suicidal thoughts and behaviors (Wolke & Lereya, 2015). The caregivers in the sample did not evidence these outcomes, which may signal difficulty to understand the impact of bullying beyond childhood. It is important to mention that, without effective intervention, the consequences of bullying can gain intensity until adolescence and in the adult phase (Wolke & Lereya, 2015).

Another fact that stands out is how the child should react when he or she is a victim of bullying. Few participants in the study consider that the child should “retaliate against aggression,” which may signal their concern about perpetuating a cycle of violence or indicate little confidence in the child’s ability to handle the situation, as many caregivers consider that preferable reactions are those that trigger adults as mediators of conflict, such as telling parents, telling school teachers, and telling school principals or coordinators. At the same time, almost all caregivers consider it is important to prevent bullying and the responsibility for this prevention must be from parents, school, and teachers. This conception can be considered positive, as studies point to the importance of supporting the child to develop problem-solving strategies, as the power imbalance typical of bullying can hinder the child’s development of coping strategies (Craig et al., 2007; Sawyer et al., 2011).

In addition, education, family values, and communication between parents and children were considered to be the main contributors to bullying prevention. The study data indicate that communication, in general, with the child, school, or teachers, was seen as the main reaction parents need to adopt when their child is an aggressor or a victim of bullying. This conception of caregivers is in line with studies that suggest that the quality of the child’s relationships with adults and the characteristics of the family climate are associated with the perpetuation of bullying (Craig et al., 2007; Shetgiri, Lin, Avila, & Flores, 2012). Communication between caregivers and children, for example, can be considered a protective factor against aggressive behaviors, as well as other factors, including involvement with the child’s social circle and academic activities (Shetgiri et al., 2012).

Another relevant finding of the study refers to the caregivers’ conception that both boys and girls can become involved in situations of aggression and victimization. Studies indicate that there are differences in the type of aggression perpetuated by boys and girls, due to differences in socialization, cultural, biological, and environmental variables (Orpinas, McNicholas, & Nahapetyan, 2015; Stubbs-Richardson, Sinclair, Goldberg, Ellithorpe, & Amadi, 2017). Bullying is a problem for both sexes, although early studies on the subject have put greater emphasis on bullying by boys. Other studies indicate that boys are more assaulted by boys and girls are mainly attacked by girls; while boys use more physical aggression, girls use indirect forms of bullying, including gossip, exclusion, and use of nicknames (Archer, 2004, Crick et al., 1999; Donoghue & Raia-Hawrylak, 2016). Most of the participants consider that older children practice more bullying. This belief also corroborates studies on aggressive behavior in childhood and adolescence, which indicate that the incidence of physical attacks tends to decrease with age, giving rise to a higher incidence of verbal aggression or aggression that impairs the victims’ social relationship (Atherton Tackett, Ferrer, & Robins, 2017; Terroso et al., 2016). These aggressions are more prevalent and detrimental as age advances, given the growing importance of social approval and reputation (Atherton et al., 2017; Weyns et al., 2017).

The understanding that bullying entails negative consequences for victims and perpetrators may explain the large number of caregivers who consider it important to punish bullying. It is important to mention that most caregivers believe that there should be a law to punish bullying situations in Brazil, but they do not know of any anti-bullying laws in the country. Although anti-bullying programs have been implemented and constantly evaluated in the USA and in European countries for decades, public policies that prioritize bullying reduction and prevention are still incipient (Borsa et al., 2015).

Most caregivers reported that their son or daughter had been a victim of bullying at school. This data alerts to the fact that bullying is still a reality present in childhood and deserves attention, given the negative impact of the phenomenon in the lives of the stakeholders (Fisher et al., 2012; Takizawa et al., 2014; Wolke & Lereya, 2015). The data also indicate that children are reporting to their parents when they are bullied, in line with the perception of caregivers that the child would tell at home about bullying if they were being victims at school. The data is also consistent with the importance the caregivers demonstrate concerning children talking to adults and the value of a communication network that involves the school, the abuser, and the victim (Shetgiri et al., 2012).

It is important to discuss that 82.5%, the vast majority of caregivers, said their child had reported witnessing bullying in school and, in contrast, 83.9% of caregivers believe that their child had never practiced bullying at school. Considering the high prevalence of victims among children, but the low prevalence of aggressors in the parents’ perception, reflection is possible on the difficulty of identifying the child as an aggressor, which can be related to both communication difficulties and difficulties in identifying certain behaviors as bullying and differentiating these behaviors from other social interactions, such as jokes or teasing, for example (Sawyer et al., 2011).

From the caregivers’ responses about the children’s reaction when they witness bullying situations, the social dynamics of bullying is evident, characterized by an interaction among offenders, victims, and witnesses who reinforce aggression (Olweus, 1993). Most respondents indicate that the child sought help from others, especially teachers and school principals, and/or defended the victim. A smaller part of the responses indicated that the child watched the aggression passively, witnessed and reinforced the aggression by finding the situation funny, or even encouraged the aggressor. On the one hand, one can consider the social desirability that makes subjects respond in a way that reveals favorable traits or present socially accepted responses. On the other hand, the result arouses reflections about the role of teachers (and principals) in the management of bullying. A robust study conducted by Veenstra, Lindenberg, Huitsing, Sainio and Salmivalli (2014) concluded that the students’ perception of the effectiveness of the teacher to intervene in bullying relates to the frequency of bullying, making them a population that needs to be present in bullying intervention and prevention programs.

## Conclusion

In this study, the conception of the caregivers on the phenomenon of bullying were analyzed, specifically in relation to its occurrence, motivations, and risks for the development, and compared to international literature. The results allowed us to understand what Brazilian caregivers think about bullying and how they act (or would act) towards situations of bullying their children experienced. The results appoint that the caregivers have good knowledge on signs and forms of coping with bullying according to the literature what may be related to the high educational level of the sample. Nevertheless, Brazilian caregivers tend not to recognize their children as potential aggressors and do not mention family as a risk factor for the occurrence of child bullying.

One of the limitations of this study is that the conception about the occurrence of bullying among the children was informed by the caregivers, not by the children themselves. In this sense, the participants’ responses depended on the quality of the information provided by the children and on the communication with the caregivers, as the experiences of aggression and victimization usually take place without the presence of the responsible caregivers (Shakoor et al., 2011). Another important limitation of the study relates to the fact that data collection was performed through social network, which may have had an impact on the sample responses and impairs its representativeness in relation to the Brazilian population. This impact may be observed on the high educational level of the sample.

Future studies should include a more heterogeneous sample and encompass, besides the caregivers’ conception, information from other informants, including teachers, and children. We can also suggest the elaboration of studies that highlight the cultural and regional aspects of the bullying experience in Brazil and its relation to familiar context. This knowledge could promote a more detailed understanding of the local context and thus ensure that possible interventions can be carried out with greater chances of effectiveness. Implications of this study for the field of psychology supports the importance of research and practice focused on the development of bullying intervention strategies and educational approaches that include caregivers. It also raises a number of questions, including the following: why do caregivers not recognize family as a risk factor or the occurrence of child bullying? And why do they tend not to recognize their children as potential aggressors? Are there communication barriers involving caregivers, child, and school?

## Abbreviations

DHC: Descending Hierarchical Classification
IRAMUTEQ: *Interface de R pour analyses Multidimensionnelles de Textes et de*
